# Disturbed Resting Functional Inter-Hemispherical Connectivity of the Ventral Attentional Network in Alpha Band Is Associated with Unilateral Spatial Neglect

**DOI:** 10.1371/journal.pone.0073416

**Published:** 2013-09-04

**Authors:** Tsutomu Sasaki, Masayuki Abe, Eiichi Okumura, Toyoji Okada, Kimito Kondo, Kensuke Sekihara, Wataru Ide, Hajime Kamada

**Affiliations:** 1 Division of Occupational Therapy, School of Health Sciences, Faculty of Medicine, Shinshu University, Matsumoto, Japan; 2 Department of Occupational Therapy, Hokuto Hospital, Obihiro, Japan; 3 Department of MEG, Yokogawa Electric Corporation, Kanazawa, Japan; 4 Department of Clinical Laboratory Medical Technology, Hokuto Hospital, Obihiro, Japan; 5 Department of Neurology, Hokuto Hospital, Obihiro, Japan; 6 Department of Systems Design and Engineering, Tokyo Metropolitan University, Tokyo, Japan; 7 Department of Neurosurgery, Hokuto Hospital, Obihiro, Japan; Hangzhou Normal University, China

## Abstract

Unilateral spatial neglect (USN) is one of the most common symptoms of right hemisphere damage; its classical symptom is that patients fail to respond to information on their left side. It has been postulated that disturbance of 2 separate attentional networks relates to the occurrence of USN. However, little is known about the underlying mechanism and neuronal substrates. In this study, we measured spontaneous neural activity by means of magnetoencephalography in 13 patients with brain damage and 5 control subjects. To study the relationship between functional connectivity at rest and severity of USN symptoms, we determined the imaginary coherence values relating to the inter-hemispherical ventral and dorsal attentional networks, as well as the clinical severity of USN using neuropsychological tests and behavioral rating scales. The present results showed that inter-hemispherical connectivity in the ventral attentional network, especially between the left and right angular gyri, detected in the alpha band is associated with the severity of USN symptoms. This may suggest that connectivity of inter-hemispherical homologous regions of the ventral attentional network in the alpha band could be one of the biomarkers of attentional network imbalance occurring in patients with USN.

## Introduction

There has been an increasing interest in understanding how the human brain works when it is at rest. In particular, the analysis of functional connectivity during rest and the associated temporal correlation of brain activities in different regions has become a popular topic of study. Abnormalities in the interactions of network components play critical roles in common neurological and psychiatric disorders such as epilepsy [1], depression [2], schizophrenia [3], dementia [4], and autism [5]. In addition, damage to specific functionally connected networks is known to lead to distinct types of cognitive dysfunction [6–9].

One of the most prominent symptoms of brain damage is unilateral spatial neglect (USN), which is well known as a common and disabling consequence of right-hemisphere damage. It is a complex syndrome characterized by a failure to attend to, look at, or respond to stimuli located on the side of the body opposite to the side of the affected hemisphere. Although a large number of studies on USN have been published, the neuronal substrates of USN are not well understood. USN occurs in about 25–30% of all stroke-affected individuals [10,11]. The most frequent sites of damage are the right temporoparietal junction [12] and the right inferior parietal [13–15], ventral frontal [16], and superior temporal gyri [17]. However, unilateral damage to subcortical regions such as the thalamus [18–20], striatum, internal capsule [20], putamen, caudate nucleus, pulvinar [21], and cerebellar region [22] have been reported to cause USN symptoms. These findings imply that structural damage of specific focal brain regions cannot fully explain the neural mechanisms underlying USN, leading to the idea that USN may be better explained by the dysfunction of distributed cortical networks that control attention [23–25].

With the recent development of brain imaging techniques, the neural mechanisms of USN have been examined from the viewpoint of brain networks implicated in the control of attention. It has been suggested that 2 attentional systems exist in the human brain: the ventral attentional network (VAN) and dorsal attentional network (DAN) [24–27]. The VAN includes the inferior frontal gyrus, ventral frontal gyrus (VFG), supramarginal gyrus (SMG), angular gyrus (AG), and superior temporal gyrus, while the DAN includes superior frontal gyrus (SFG), superior parietal lobule, and middle temporal gyrus. In most cases with USN, lesions appear to cluster around a large perisylvian network in the right hemisphere [12,23,28]. Using diffusion tensor imaging tractography, Urabanski et al. [29] identified that damage to clusters in the perisylvian white matter lead to USN. He et al. [30] demonstrated that disrupted functional connectivity in the VAN is manifested in cases of USN, especially during the acute phase. With respect to the DAN, it has been reported that functional connectivity between inter-hemispheric homologous regions in the DAN, under task-driven conditions [30,31], as well as under resting conditions [32], is associated with USN symptoms.

The majority of studies demonstrating a relationship between functional connectivity and USN symptoms have employed functional magnetic resonance imaging (MRI). An important limitation of this technique is that it measures slow fluctuations in the blood oxygen level dependent signal, an indirect measure of neural activity in the brain [33]. On the other hand, electroencephalography and magnetoencephalography (MEG) directly measure electrophysiological brain activity. Furthermore, the high temporal resolution of electroencephalography and MEG allow for the separation of neuronal activity into oscillatory components that reflect distinct biophysical properties [34]. In fact, several studies have revealed relationships between neural oscillation and brain function [35–37]. However, to the best of our knowledge, no study to date has directly measured both neural activity and functional connectivity in patients with USN. The purpose of the present study was to 1) use MEG to measure neural activity in patients with USN during rest and 2) evaluate the relationship between functional connectivity and USN severity.

## Materials and Methods

### Subjects

Thirteen stroke patients with right-hemisphere damage (mean age 65.1 ± 11.8 (years ± SD) in the range of 38–82 years; 7 men and 6 women) and 5 healthy volunteers (mean age 26.0 ± 1.3 (years ± SD) in the range of 25–28 years; 3 men and 2 women) participated in this study. The demographic and clinical characteristics of the participants are summarized in Table 1. All patients received standard therapy at the stroke unit during the acute phase and individually tailored multidisciplinary rehabilitation programs during the subacute, recovery, and chronic phases. All were right-handed as assessed by the Edinburgh Inventory [38]. Subjects were in good health and had no history of neurological or psychiatric disease. The present study was conformed to the ethical principles of the Helsinki Declaration, and approved by the Ethics Committee of Shinshu University and by the Ethics Committee of Hokuto Hospital. Written informed consent was obtained from each subject.

**Table 1:** Clinical data.

					Months		USN	BIT	
					from		Index	3 types	CBS
Case	Sex	Age	LQ	Etiology	onset	MMSE	(/96)	(/66)	(/30)
USN(+) 1	M	73	100	Inf	8	21	42	39	3
USN(+) 2	W	78	100	Inf	9	15	56	44	12
USN(+) 3	M	82	82	Inf	1	13	36	26	10
USN(+) 4	M	38	100	Hemo	2	17	29	24	5
USN(+) 5	W	79	80	Inf	4	23	35	25	10
USN(+) 6	W	65	100	Inf	4	25	12	9	3
USN(+) 7	W	63	100	Inf	3	29	16	1	15
USN(+) 8	M	71	100	Inf	1	29	5	1	4
USN(-) 1	M	56	100	Inf	1	29	0	0	0
USN(-) 2	W	60	100	Hemo	1	24	0	0	0
USN(-) 3	M	50	100	Inf	2	27	0	0	0
USN(-) 4	W	65	100	Inf	3	29	0	0	0
USN(-) 5	M	66	100	Hemo	4	27	0	0	0
Normal 1	W	25	98	-	-	30	0	0	0
Normal 2	M	25	100	-	-	30	0	0	0
Normal 3	M	27	100	-	-	30	0	0	0
Normal 4	W	25	99	-	-	30	0	0	0
Normal 5	M	28	100	-	-	30	0	0	0

Inf: infarction; Hemo: hemorrhage; LQ: Laterality Quotient [38] ; MMSE: Mini-Mental State Examination [39] ; BIT: Behavioural Inattention Test [40] ; CBS: Catherine Bergego Scale [41] .

### Clinical assessment

All subjects were evaluated by means of the following 4 neuropsychological tests: i) the Mini-Mental State Examination scored from 0 (severe) to 30 (mild) [39], ii) the Star Cancellation in the Behavioural Inattention Test (BIT) [40] scored from 0 (mild) to 54 (severe), iii) the Daisy Copying (BIT) scored from 0 (mild) to 3 (severe), iv) the Line Bisection (BIT) scored from 0 (mild) to 9 (severe). All subjects were also scaled on their behavioral attentional disturbance in daily living using the Catherine Bergego Scale (CBS) scored from 0 (mild) to 30 (severe) [41]. To confirm whether subjects exhibit USN and to measure the severity of USN, we defined the USN index as the total score of the Star cancellation, the Daisy Copying, the Line Bisection, and the CBS tests, which totally ranges from 0 (mild) to 96 (severe). To control for the impact of right hemisphere damage per se, subjects with brain damage were classified into USN(+) and USN(-) on the basis of the USN index: USN(+) if the USN index is above 0, USN(-) if the index scores 0. As shown in Table 1, USN symptoms were evident in 8 out of the 13 patients with brain damage. Subjects were analyzed in 3 groups: 1) Normal controls; 2) USN(-) patients with brain damage but no USN symptoms; and 3) USN(+) patients with brain damage and USN symptoms.

### Structural MRI

**Figure 1:**
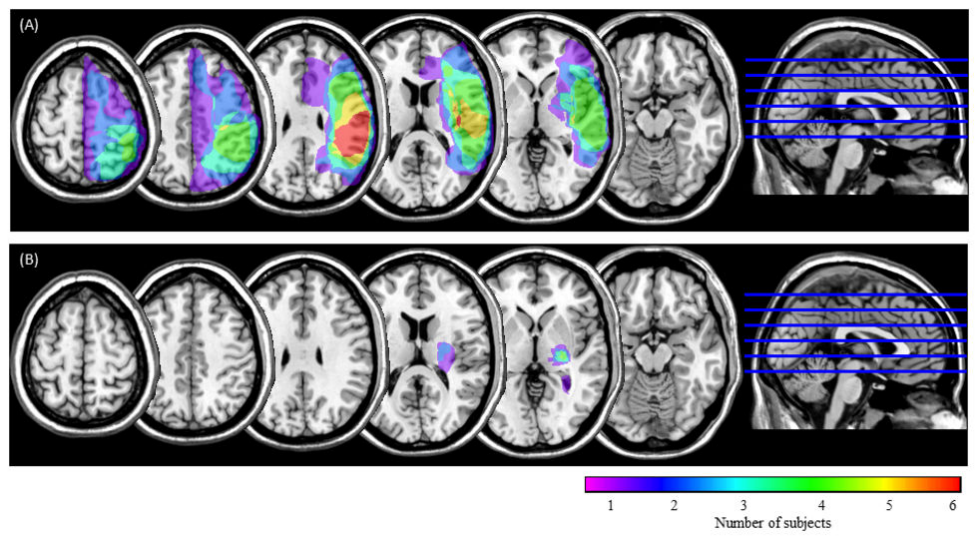
Lesion overlap maps. USN(+) group (A) and USN(-) group (B). Horizontal sections through a template brain show the frequency of damage for each voxel. The color scale indicates the increasing frequency of overlapping lesions from violet (n = 1) to red (n = 6).

Imaging data were acquired on a Signa 3.0 Tesla system (GE Healthcare). High-resolution structural images were acquired with a three-dimensional fast spoiled gradient-recalled-echo T1-weighted sequence (repetition time: 9.5 msec; echo time: 3.9 msec; flip angle: 13°; field of view: 240 mm; slice thickness: 1.6 mm; matrix: 288 × 288; 128 slices). For patients with stroke, we drew lesion overlap maps from the T1-weighted images (Figure 1). Lesion extent was determined for each patient by selecting brain scans that showed the greatest extent of damage and drawing the lesion borders directly onto the original images, using the MRIcron software [42] available online (http://www.nitrc.org/projects/mricron). All lesion maps were double-checked by a neurologist or a therapist trained to read brain scans.

### MEG recording

Magnetic fields were measured in a 160-channel whole-head-type gradiometer system (MEGvision PQA1160C; Yokogawa Electric Corporation, Japan). MEG data were sampled at 1000 Hz per channel (band pass 0.16–200 Hz and notch 50 Hz) with the subjects in a supine position with eyes closed for 10 minutes. During the MEG recordings, subjects were instructed to close their eyes and reduce eye movements, but to remain awake as much as possible. During the recordings, the investigator and MEG technician checked the on-line signal for visual signs of drowsiness (e.g., slow eye movement activity) and observed the patients using a video monitor. Each subject’s head position relative to the sensor array was determined before and after the MEG recording.

### Data analysis

#### General

Data were analyzed using Matlab 7.13 (MathWorks, Natick, MA) and custom scripts for general technical computing and source reconstruction, the FieldTrip open source Matlab toolbox [43] (http://www.ru.nl/fcdonders/fieldtrip) for functional connectivity analysis, component analysis, SPM8 (Wellcome Department of Cognitive Neurology, London, UK, http://www.fil.ion.ucl.ac.uk/spm/software/spm8/) for spatial normalization, and Statistics toolbox 7.6 (MathWorks, Natick, MA) for statistical analysis. The SPSS 17.0 was also used for statistical analysis.

#### Preprocessing

First, MEG data were subjected to principle component analysis (PCA) and typical noise components (e.g. magnetized metal artifacts) were removed using visual inspection. After that, we defined trials of interest from the continuous MEG data. Trials were defined as continuous data segments of two-second frame length in steps of 1 second, excluding trials with magnetic flux in any channel that exceeded 2000 fT. The number of analyzed trials, 140 trials, was equalized among subjects.

#### Region of interest and network nodes

For functional connectivity analysis, first, we selected 16 regions of interest (ROIs) involved in attentional networks based on the previous studies [24–27,30–32]: the DAN consisting both the SFG, superior parietal lobule, and middle temporal region, and the VAN consisting of both the VFG, the inferior frontal gyrus, the SMG, the AG, and the superior temporal gyrus. Secondly, these 16 ROIs were spatially normalized according to the Montreal Neurological Institute coordinate system, and the structural volumes of ROIs were obtained using WFU-PickAtlas 3.0.3 software (ANSIR Laboratory, Wake Forest University School of Medicine) [44,45]. The ROI labels used were from the automated anatomical labeling atlas of 116 segmented structures [46]. Finally, the nodes were generated by means of down-sampling these ROI three-dimensional images from 2-mm to 10-mm spacing. 388 nodes in 16 ROIs were obtained. The number and position of nodes in each ROI is summarized in Table S1 and Figure S1, respectively.

#### Source-space coherence analysis

Coherence is a widely used representative measure, and we adopted the imaginary part of coherence (imaginary coherence; IC) to remove the spurious coherence caused by leakage associated with our imaging algorithm [47,48]. IC exploits the fact that phase similarities among time series arising from a common reference or volume conduction occur with zero time delay. Thus, by omitting the real component of coherence, which mostly contains similarities with zero time lag, we removed suspect associations. By limiting our analysis to the IC, our goal was to reveal only true interactions between brain areas occurring with a certain time lag [49].

**Figure 2:**
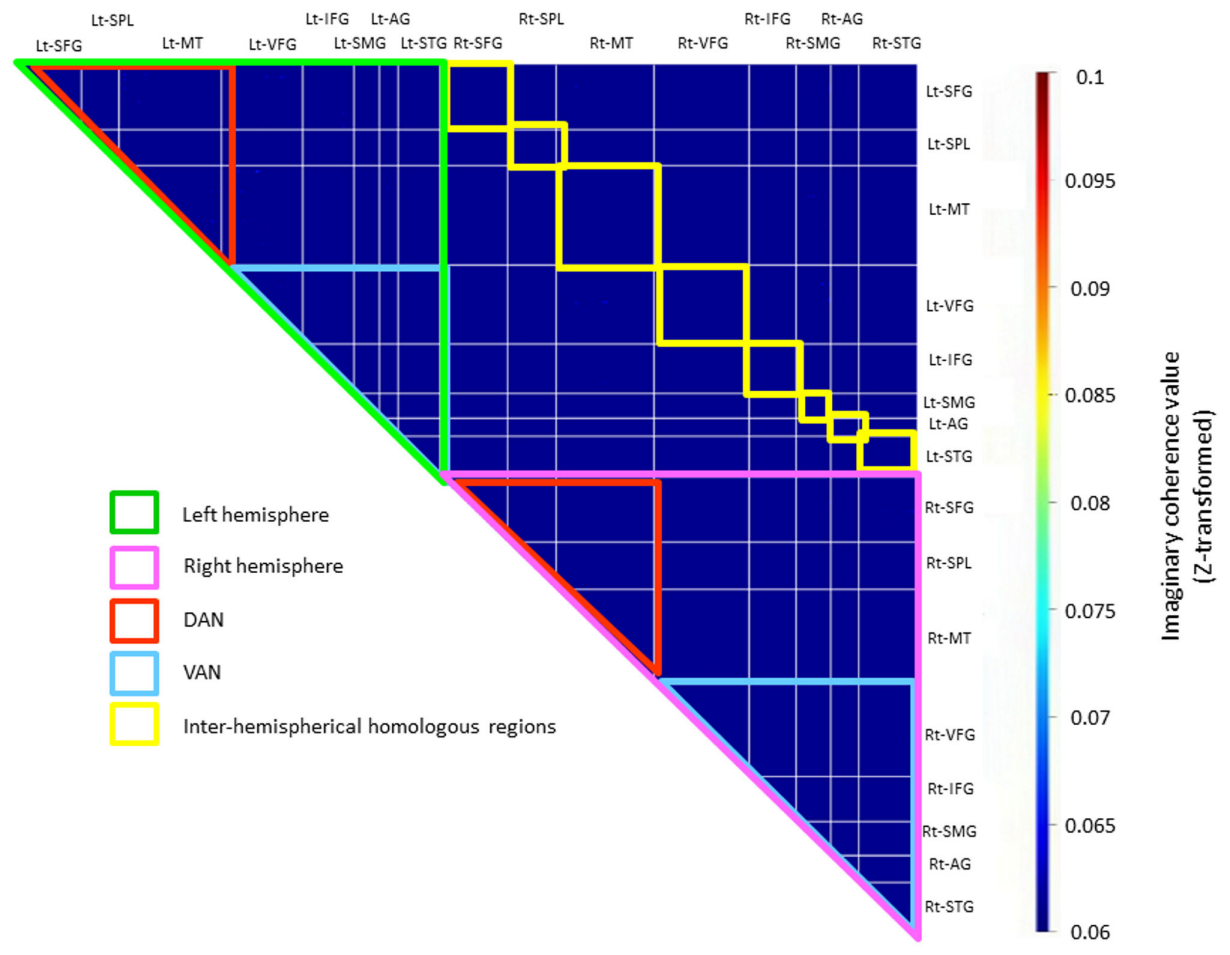
Illustration of IC matrix. The color scale indicates magnitude of imaginary coherence (Z-transformed). Area surrounded by green dashed line, and by pink dashed line indicates IC of left hemisphere and right hemisphere, respectively. Area surrounded by red dashed line and by blue dashed line indicates IC of the DAN and the VAN, respectively. Yellowed areas indicate inter-hemispherical homologous regions. SFG: superior frontal gyrus; SPL: superior parietal lobule; MT: middle temporal region; VFG: ventral frontal gyrus; IFG: inferior frontal gyrus; SMG: supramarginal gyrus; AG: angular gyrus; STG: superior temporal gyrus.

Source-space coherence analysis was performed the following procedure. First, the node locations on individual MRI coordinates were generated by using predefined ROIs (Table S1 and Figure S1) and the warping parameters calculated by SPM8 with MRI-T1 template and individual MRI-T1 images. Seconds, the time course for each node was calculated by adaptive spatial filtering [50], using a single spherical volume conductor model based on the individual MRI T1-image. To avoid potential de-ranking after PCA noise rejection, we performed the Tikonov regularization for the covariance matrix [51,52]. Next, IC of all node combinations were calculated using Fourier transform with Hanning window. The absolute value of IC was used rather than coherency because we were interested in the magnitude of connectivity at each voxel rather than in the directionality of the information flow [53]. Then, we calculated average coherence for 6 frequency bands: delta, 1–4 Hz; theta, 4–8 Hz; alpha, 8–13 Hz; beta, 13–30 Hz; low gamma, 30–50 Hz; and high gamma, 50–100 Hz [27,54–57]. The connectivity (that is, IC) at each frequency of interest was estimated by averaging across all its Fisher’s Z-transformed (arctanh(IC); the inverse hyperbolic tangent of IC) connections [47]. Then we obtained individual subjects’ IC matrices and grand-averaged (on Z-transformed space) IC matrices across subjects for each group (see Figure 2). Finally, the ROI IC values were calculated for each subject by averaging across voxel pairs within each ROI. These ROI IC values were used for correlation analysis between IC and USN index.

#### Correlation between IC and USN index

To determine whether functional connectivity predicts clinical severity of USN, we performed regression analysis. In this analysis, normal controls were excluded to reveal the influence of brain damage on functional connectivity relating to USN symptom more clearly.

## Results

### IC among groups at each frequency band

**Figure 3:**
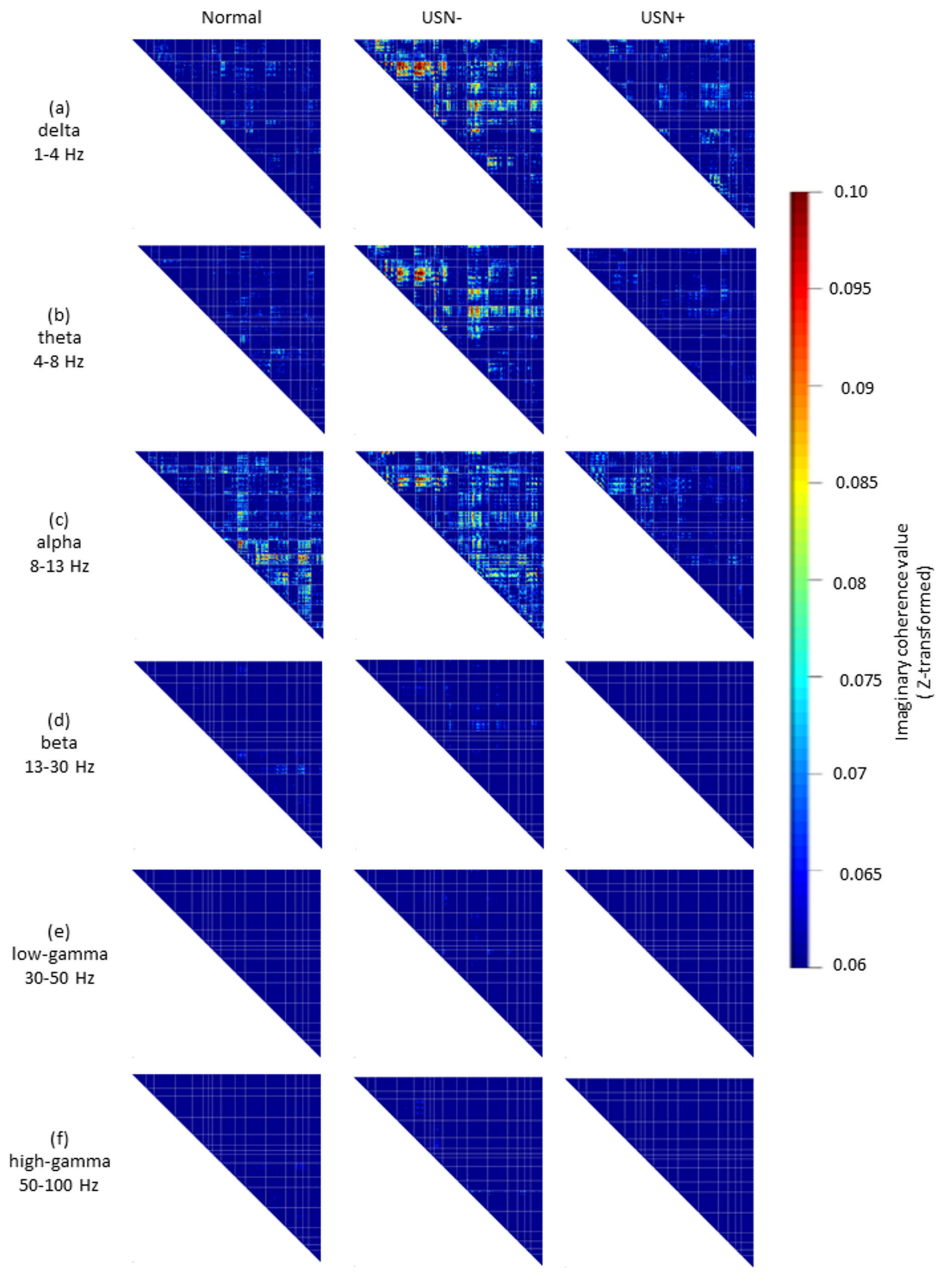
IC matrices among groups at each frequency band. The patterns of IC matrices in the delta (a) and theta band (b) were similar. The matrices in the beta band (d) and low-/high-gamma (e, f) band were also similar. Unlike the IC matrices in other frequency bands, the IC matrices in the alpha band (c) were considerably different among the groups. The color scale indicates imaginary coherence value (Z-transformed).

Figure 3 showed that the IC matrices among groups in the delta and theta band were strikingly similar. For these bands, the USN(-) group seemed to exhibit higher connectivity between the left DAN/left VAN and between the /left/right hemisphere than did the other 2 groups (Figure 3a, 3b). ANOVA revealed, however, that no statistical difference was found among groups for the comparison between the left DAN and the left VAN (F_2,15_ = 2.828, p > 0.1 for delta band: F_2,15_ = 2.914, p > 0.1 for theta band), and between left hemisphere and right hemisphere (F_2,15_ = 1.407, p > 0.1 for delta band: F_2,15_ = 1.099, p > 0.1 for theta band). The matrices among groups in the beta band and low-/high gamma band were also similar. For these 3 bands, each group seemed to exhibit lower connectivity than in other oscillatory bands (Figure 3d, 3e, 3f). The patterns of IC maps in the alpha band seemed to be considerably different from that of other frequency bands. Each group exhibited different IC pattern. For normal group, IC within right hemisphere seemed to be higher than within left hemisphere, however, statistical analysis showed no difference (t(8) = -1.699, p > 0.1). For the USN(-) group, although IC within left hemisphere seemed to be higher IC than within right hemisphere, no statistical difference was found(t(8) = -0.648, p > 0.1). For the USN(+) group, IC within left hemisphere seemed to be higher than within right hemisphere, however, no statistical difference was found (t(14) = 0.740, p > 0.1) (Figure 3c).

Based on previous findings [30–32], which suggested connectivity between inter-hemispherical homologous regions in attentional networks, our goal was to determine whether inter-hemispherical connectivity between homologous regions in the DAN as well as in the VAN (e.g., left and right SFG) contribute to USN symptoms.

**Figure 4:**
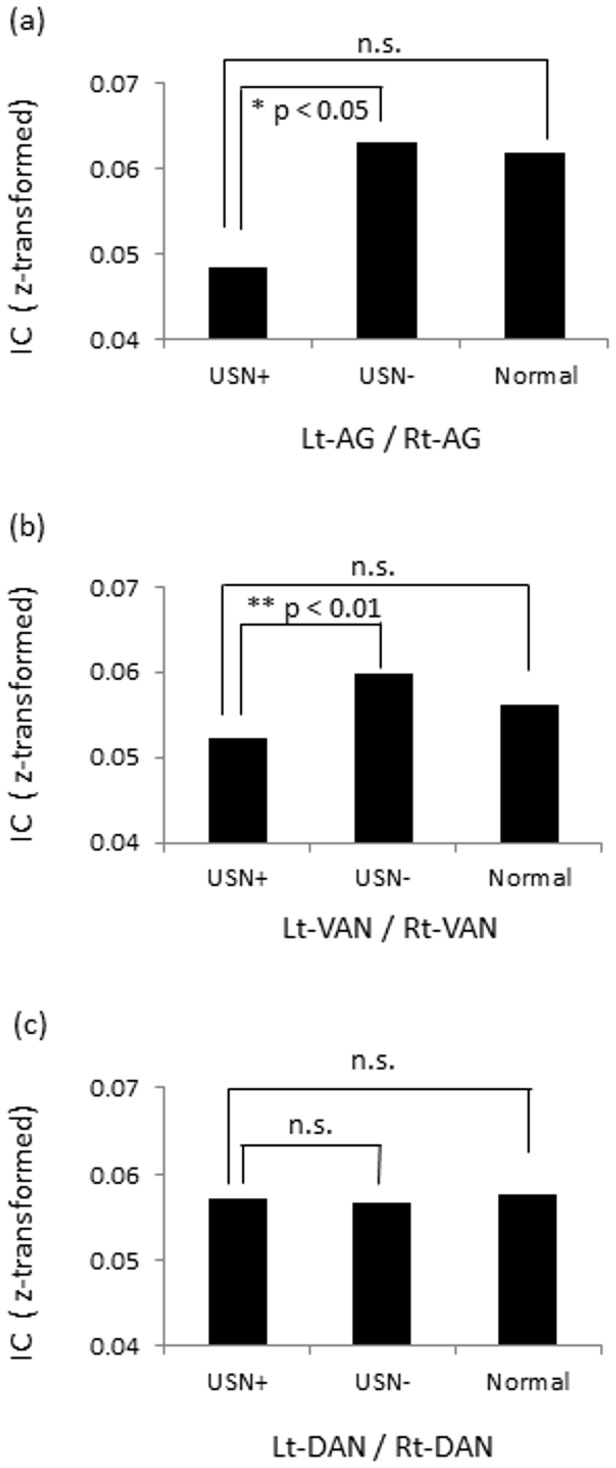
Comparisons of IC of inter-hemispheric attentional regions in the alpha band (8–13 Hz). (a) In left/right AG, the USN(+) group showed lower IC than the USN(-) group (uncorrected p < 0.05). (b) In left/right VAN, the USN(+) group showed lower IC than USN(-) (uncorrected p < 0.01). (c) In left/right DAN, no statistical difference was found among the groups.

For the delta band, ANOVA revealed statistically different IC between the left and right SFG among the groups (F_2,15_ = 4.764, p < 0.05). Post hoc analysis confirmed that the USN(+) group exhibited greater IC than the normal group (uncorrected p < 0.05). The IC between the left and right VFG also showed significantly different patterns among the groups (F_2,15_ = 3.964, p < 0.05). Post hoc comparison confirmed that compared to the normal group, the USN(+) group exhibited greater IC (uncorrected p < 0.05). In the theta and beta bands, no differences between inter-hemispherical homologous regions were found among the groups. For the alpha band, there was a significant difference between the left/right AG among groups (F_2,15_ = 5.105, p < 0.05), and post hoc comparisons confirmed that the USN(+) group showed lower IC than the USN(-) group (uncorrected p < 0.05), not but compared to the normal group (Figure 4a). We also compared IC between the left and right VAN, as well as between the left and right DAN, in the alpha band among the groups. Analysis showed a statistical difference among the groups for the VAN (F_2,87_ = 5.566, p < 0.01), and post hoc comparisons confirmed that compared to the USN(-) group, the USN(+) group showed lower IC (uncorrected p < 0.01) but no difference was found between the USN(+) group and the normal group (uncorrected p > 0.05) (Figure 4b), while for the DAN, there was no statistical difference among groups (F_2,51_ = 0.034, p > 0.10) (Figure 4c). For the gamma band, ANOVA revealed a significant difference among groups in the IC between the left and right AG in the lower range (30–50 Hz) (F_2,15_ = 4.227, p < 0.05) and between the left and right SMG in the higher range (50–100 Hz) (F_2,15_ = 4.404, p < 0.05). Post hoc comparisons confirmed that the USN(+) group exhibited lower IC than the USN(-) group in both ranges (each at uncorrected p < 0.05). We also calculated IC of the inter-hemispherical attentional network for the low-gamma band. Analysis revealed a statistical difference among the groups for the VAN (F_2,87_ = 6.045, p < 0.01), and post hoc comparisons confirmed that compared to the USN(-) group, the USN(+) group showed lower IC (uncorrected p < 0.01), while for the DAN, there was no statistical difference among groups (F_2,51_ = 0.949, p > 0.10).

### Correlation between IC and USN index

**Figure 5:**
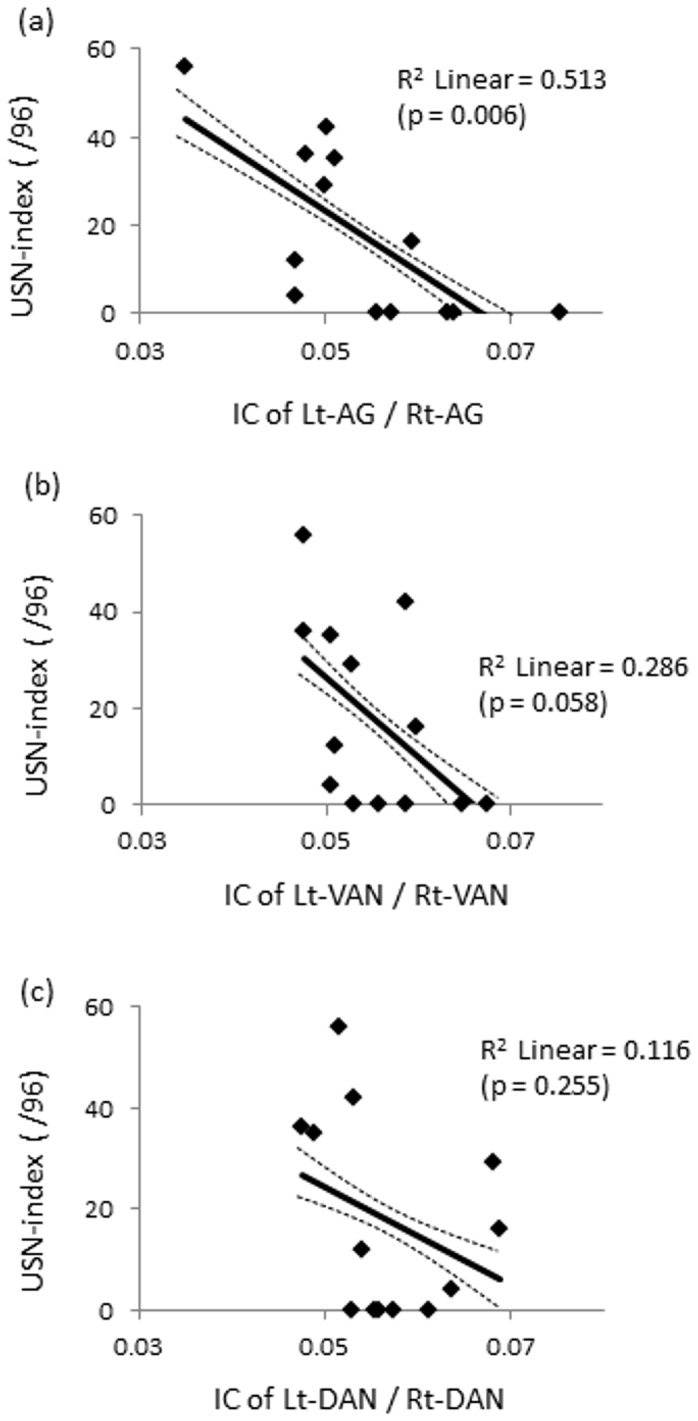
Correlations between imaginary coherence and USN index in the alpha band (8–13 Hz). Scatter plot of data for 13 patients with brain damage. (a) A significant correlation with IC of the left/right AG and USN index was found (F(1,11) = 11.597, p = 0.006, r^2^ = 0.513). (b) A correlation with IC of the left/right VAN and USN index showed a trend for significance (F(1,11) = 4.403, p = 0.058, r^2^ = 0.286). (c) but no significance was found in the left/right DAN (F(1,11) = 1.442, p = 0.255, r^2^ = 0.116). The solid line and dotted line represents regression line, 95% confidence intervals for the line of best fit, respectively.

To determine whether the connectivity between inter-hemispherical homologous regions in attentional networks predicts USN severity, we performed regression analysis. For the delta band, a significant correlation was found between the connectivity of the left/right SFG and USN index (F(1,11) = 10.773, p = 0.007, r^2^ = 0.495). For the theta band, no significant relation was found. For the alpha band, a significant correlation was found for the left/right AG and USN index (F(1,11) = 11.597, p = 0.006, r^2^ = 0.513) (Figure 5). We also performed regression analysis for the DAN and VAN in the alpha band, demonstrating that correlation between the VAN and USN index showed a trend for significance (F(1,11) = 4.403, p = 0.058, r^2^ = 0.286), while correlation in the DAN did not reach significant level (F(1,11) = 1.442, p = 0.255, r^2^ = 0.116) (Figure 5). For other frequency bands, i.e., the beta band, the low-gamma band, and the high-gamma band, no significant relation was found excepting the relation between the IC of the left/right STG and USN index in the high-gamma band (F(1,11) = 5.290, p = 0.04, r^2^ = 0.325).

## Discussion

This is the first report demonstrating a relationship between MEG measures of resting-state functional connectivity in specific oscillatory bands and clinical symptoms in patients with USN. Carter et al. [32] previously reported a relationship between resting functional connectivity and USN symptoms, in which inter-hemispherical functional connectivity predicted USN symptoms. However, they did not shed light on the relevance of their results to different frequencies of neural oscillation. Furthermore, they did not implicate the VAN in their findings. We have extended their results by demonstrating that patient with USN had lower inter-hemispheric connectivity in the alpha band for the VAN, especially the AG, and that IC value for these regions was correlated with severity of USN.

Two attentional networks, the DAN and VAN, are thought to be functionally heterogeneous, but to interact with each other. The DAN is involved in shifting and maintaining attention [58], goal-directed stimulus, and response selection [59] and contains visuotopic maps of contralateral space [60]. The DAN is usually intact in patients with USN [61,62]. On the other hand, the VAN is damaged in a majority of patients with neglect [61,62], and appears to be specialized for redirecting the DAN to novel and behaviorally relevant stimuli [31]. The VAN is also involved in non-spatial processes such as vigilance [63] as well as detection [64,65]. Direct damage of ventral regions, such as that observed in most of the patients in this study, causes a malfunctioning of non-spatial processes and abnormal interactions between the VAN and DAN. Finally, the occurrence of inter-hemispherical imbalance contributes to spatial neglect [24,25]. Based on these findings, the most plausible explanation of the present results may be that reduced connectivity through the corpus callosum between left and right AG [66] resulted in the dysfunction of the DAN; the impaired functioning of the DAN, in turn, interfered with patients’ abilities to shift and maintain attention (i.e., spatial neglect).

One of the most fascinating results of this study is that the disturbed connectivity of the attentional network discussed above was most striking in the alpha band. Notably, patients with USN also showed lower inter-hemispherical connectivity of the VAN in the low-gamma band; however, the IC values in the low-gamma band were not correlated with USN severity. One reason for the strength of our observation in the alpha band is that the alpha band signal is robust during rest [53] and has the highest signal-to-noise ratio of all the frequency bands [67]. In addition, as demonstrated by Hinkley et al., reliability of measuring the alpha band signal is high both within-sessions and cross-sessions as compared to that of measuring signals at other frequency bands [68]. Furthermore, it has been hypothesized that the alpha band may play a role in attentional processing [69–71], the visual network [72], and in inter-hemispherical network balance [73]. On the other hand, it has been reported that the gamma band may play a role in network modulation in the active condition [74,75], whereas slow waves, including those in the delta and theta range, may be followed by brain damage itself [76,77]. Accordingly, we can infer that the alpha band IC between the left and right VAN, especially in the AG, could be one of the biomarkers of attentional network imbalance occurring in patients with USN.

Our results are also consistent with previous findings that spontaneous neural activity at rest predicts clinical variables such as epilepsy [1], depression [2], schizophrenia [3], dementia [4], and autism [5]. Resting functional connectivity relates not only to symptoms, but also to task performance dedicated to a given cognitive function [78–80]. As pointed out in Rosazza et al. [81], resting neural activity consumes the majority of brain energy [82] and supports neural signaling processes subserving the integration of information originating from internal as well as external phenomena. With respect to the present results, a change in baseline communication, in the left/right VAN, especially in the alpha band, may significantly affect the way these regions are recruited and how they communicate in a range of situations.

Although the small size of our subject cohort may limit the statistical power of our results, we feel confident in concluding that disturbed alpha band left/right VAN connectivity, especially in the AG, is correlated with severity of USN symptoms. However, considering that individual variations of functional network in healthy subjects [25] and of brain activities in patients with USN [83], it is obvious that we should collect data from additional subjects with and without USN as well as age-matched healthy controls to elaborate present findings. Another limitation of this study is that we did not focus on the networks other than attentional networks. Therefore, we could not specify the network unrelated to attentional control as well as the relationship between these networks. Further study should address issues of interactions across bands, power analysis [35], non-stationary analysis [27], and network changes after treatment [84] with larger populations of subjects.

## Supporting Information

Figure S1 **Representations of position of 388 nodes.** (a) top view, (b) front view, (c) back view, (d) left side view, (e) right side view. Light-blued dots represent SFG (superior frontal gyrus), dark-blue represents SPL (superior parietal lobule), blue represents MT (middle temporal region). Red, orange, pink, yellow, and green represents VFG (ventral frontal gyrus), IFG (inferior frontal gyrus), AG (angular gyrus), SMG (supramarginal gyrus), STG (superior temporal gyrus), respectively.
(TIF)

Table S1 **The number of nodes (voxels) in each ROI.** SFG: superior frontal gyrus; SPL: superior parietal lobule; MT: middle temporal region; VFG: ventral frontal gyrus; IFG: inferior frontal gyrus; SMG: supramarginal gyrus; AG: angular gyrus; STG: superior temporal gyrus.
(DOCX)
