# Supplementary figures and images for: Disturbed Resting Functional Inter-Hemispherical Connectivity of the Ventral Attentional Network in Alpha Band Is Associated with Unilateral Spatial Neglect
Source: PLoS One. 2013 Sep 4;8(9):e73416. doi: 10.1371/journal.pone.0073416 (PMC3762777; doi:10.1371/journal.pone.0073416)

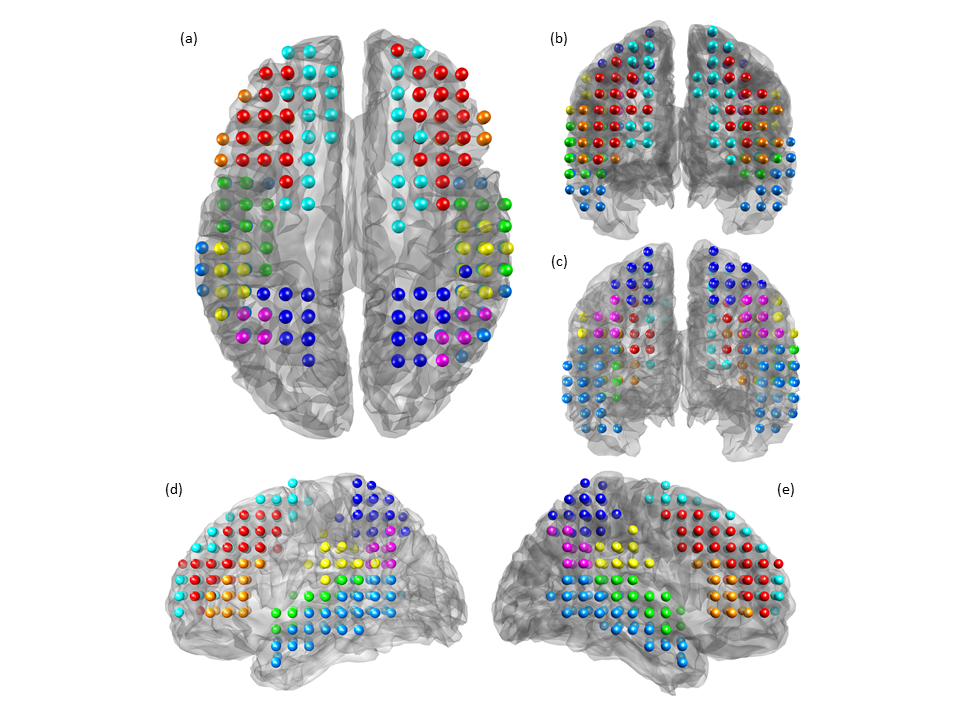

Supplement: Figure S1 — Representations of position of 388 nodes. (a) top view, (b) front view, (c) back view, (d) left side view, (e) right side view. Light-blued dots represent SFG (superior frontal gyrus), dark-blue represents SPL (superior parietal lobule), blue represents MT (middle temporal region). Red, orange, pink, yellow, and green represents VFG (ventral frontal gyrus), IFG (inferior frontal gyrus), AG (angular gyrus), SMG (supramarginal gyrus), STG (superior temporal gyrus), respectively. (TIF) [file pone.0073416.s001.tif]
