# Supplementary material for: Disturbed Resting Functional Inter-Hemispherical Connectivity of the Ventral Attentional Network in Alpha Band Is Associated with Unilateral Spatial Neglect
Source: PLoS One. 2013 Sep 4;8(9):e73416. doi: 10.1371/journal.pone.0073416 (PMC3762777; doi:10.1371/journal.pone.0073416)
Supplement: Table S1 — The number of nodes (voxels) in each ROI. SFG: superior frontal gyrus; SPL: superior parietal lobule; MT: middle temporal region; VFG: ventral frontal gyrus; IFG: inferior frontal gyrus; SMG: supramarginal gyrus; AG: angular gyrus; STG: superior temporal gyrus. (DOCX) [file pone.0073416.s002.docx]

**Supporting information**

**Table S1. The number of nodes (voxels) in each ROI**.

|  | Left hemisphere | Right hemisphere |
| --- | --- | --- |
| SFG | 29 | 29 |
| SPL | 16 | 21 |
| MT | 44 | 42 |
| VFG | 35 | 41 |
| IFG | 22 | 20 |
| SMG | 11 | 15 |
| AG | 8 | 12 |
| STG | 18 | 25 |

SFG: superior frontal gyrus; SPL: superior parietal lobule; MT: middle temporal region; VFG: ventral frontal gyrus; IFG: inferior frontal gyrus; SMG: supramarginal gyrus; AG: angular gyrus; STG: superior temporal gyrus.
